# The Importance of Non-Diffusional Factors in Determining Photosynthesis of Two Contrasting Quinoa Ecotypes (*Chenopodium quinoa* Willd.) Subjected to Salinity Conditions

**DOI:** 10.3390/plants10050927

**Published:** 2021-05-06

**Authors:** José Delatorre-Herrera, Karina B. Ruiz, Manuel Pinto

**Affiliations:** 1 Doctoral Program in Agriculture for Arid-Desert Environments, Faculty of Renewable Natural Resources, Desert Agriculture Area, Universidad Arturo Prat, Iquique 1100000, Chile; 2 Facultad de Ciencias de la Salud, Universidad Arturo Prat, Iquique 2120, Chile; karuiz@unap.cl; 3 Plant Physiology Laboratory, Institute of Agronomic and Veterinary Sciences, Universidad de O´Higgins, Rancagua 2820000, Chile; manuel.pinto@uoh.cl

**Keywords:** Na^+^, K^+^, CO_2_ assimilation, stomatal restrictions, non-diffusional, diffusional, RubisCO activity

## Abstract

The broad distribution of quinoa in saline and non-saline environments is reflected in variations in the photosynthesis-associated mechanisms of different ecotypes. The aim of this study was to characterize the photosynthetic response to high salinity (0.4 M NaCl) of two contrasting Chilean genotypes, Amarilla (salt-tolerant, *salares* ecotype) and Hueque (salt-sensitive, coastal ecotype). Our results show that saline stress induced a significant decrease in the K^+^/Na^+^ ratio in roots and an increase in glycine betaine in leaves, particularly in the sensitive genotype (Hueque). Measurement of the photosynthesis-related parameters showed that maximum CO_2_ assimilation (A_max_) in control plants was comparable between genotypes (ca. 9–10 μmol CO_2_ m^−2^ s^−1^). However, salt treatment produced different responses, with A_max_ values decreasing by 65.1% in the sensitive ecotype and 37.7% in the tolerant one. Although both genotypes maintained mesophyll conductance when stomatal restrictions were removed, the biochemical components of Amarilla were impaired to a lesser extent under salt stress conditions: for example, the maximum rate of ribulose-1,5-bisphosphate carboxylase/oxygenase (RubisCO; V_cmax_) was not as affected in Amarilla, revealing that this enzyme has a higher affinity for its substrate in this genotype and, thus, a better carboxylation efficiency. The present results show that the higher salinity tolerance of Amarilla was also due to its ability to control non-diffusional components, indicating its superior photosynthetic capacity compared to Hueque, particularly under salt stress conditions.

## 1. Introduction

At present, about one-third of the world’s irrigated land [1] is affected by salinity, which reduces plant growth and crop yield [2]. As the main food crops are rather sensitive to this stress, tolerance to salinity has become an important agronomical trait for breeders, physiologists, and agronomists [3]. The salinity of soils is predominantly caused by salt transported by irrigation water [4]. On the other hand, due to climate change, well water is becoming the main water resource for irrigation in many parts of the world, and several studies have indicated that this method is linked to potential risks of soil salinization [5,6,7]. An increase in the soil concentration of Na^+^ and other ions, such as Ca^2+^, Cl^−^, and K^+^, causes a decrease in the soil water potential, which limits the water absorbed by roots and induces water stress [8]. Specific ion toxicities are due to the accumulation of sodium, chloride, and/or boron in the tissue of transpiring leaves to damaging levels. The accumulation of injurious ions may inhibit photosynthesis and protein synthesis, inactivate enzymes, and damage chloroplasts and other organelles [9]. Under normal physiological conditions, plants maintain a high cytosolic K^+^/Na^+^ ratio. Given the difference in negative membrane potential at the plasma membrane (–140 mV), a rise in extracellular Na^+^ concentration will establish a large electrochemical gradient that favors the passive transport of Na^+^ into cells through the activation of K^+^ transporters/channels and through non-selective channels that are sensitive to Ca^2+^. The permeation of Na^+^ via voltage-independent cation (VIC) channels is inhibited by an increase in extracellular Ca^2+^ concentration [10]. Overaccumulation of Na^+^ in the cytosol inhibits protein synthesis, enzyme activity [1], and many photosynthetic processes [11,12]. Therefore, maintaining its water supply and excluding Na^+^ from photosynthetic organs are crucial mechanisms used by tolerant plants to ensure an adequate rate of carbon fixation under salt stress [13]. It is well known that a reduction in stomatal conductance negatively affects the CO_2_ assimilation rate as well as the water balance in leaves [3,14].

Excess salt affects plant growth due to an increase in soil osmotic pressure and interference with plant nutrition. A high salt concentration in the soil solution reduces the ability of plants to acquire water, which is referred to as the osmotic or water-deficit effect of salinity [9]. A decrease in CO_2_ assimilation is a widely reported effect of salt [15]. In many cases, this decrease is associated with the negative effect of salinity on diffusional mechanisms. These mechanisms depend on the gradient between the external and internal CO_2_ concentrations (mesophyll and chloroplasts), on the stomatal conductance in the gaseous phase (g_s_), and on the mesophyll conductance in the liquid phase (g_m_) [15,16,17,18].

However, salt also affects non-diffusional mechanisms, which are typically associated with electron transport and the activity of enzymes in carboxylation cycles; key enzymes in these processes include ribulose-1,5-bisphosphate carboxylase/oxygenase (RubisCO) [19,20] and those involved in the regeneration of ribulose-1,5-bisphosphate (RuBP) [21,22]. 

Most authors agree that diffusional factors are predominant in affecting the CO_2_ assimilation rate [15,23]. For example, Flexas et al. [24] reported that the conductance of the mesophyll in grapevine was strongly correlated with the rate of photosynthesis. In olive trees (*Olea europaea*), this occurs in the leaves of stressed plants with a conductance greater than 50 µmol m^−2^ s^−1^ [22]. According to the aforementioned study, the combined reduction in stomatal and mesophyll conductance in different salt-stressed olive cultivars increases the difference in CO_2_ concentration between the environment and chloroplasts. These results indicate that the low concentration of CO_2_ in chloroplasts caused by decreased stomatal and mesophyll conductance is the main limiting factor of photosynthesis.

Quinoa (*Chenopodium quinoa* Willd.) has evolved a number of adaptive responses to saline stress conditions, such as the increased elasticity of cell walls, low water potential, osmotic adjustment, reduction in the foliar area, the presence of *papillae* with calcium oxalate, the production of organic solutes [25,26,27,28], the adaptation of physiological functions such as photosynthesis [2,29], the regulation of the water status [29], ionic partitioning [30,31], and changes in stomatal conductance [3,16,27].

There are an increasing number of studies on CO_2_ assimilation in quinoa [2,16,26,32,33], although few have characterized the importance of diffusional and non-diffusional mechanisms. This species is particularly diverse at the genetic level [34], a consequence of its broad distribution in both saline and non-saline environments. This diversity could allow some ecotypes to thrive under saline stress conditions due to improved rates of photosynthesis [30].

Thus, it is possible to postulate that saline-tolerant ecotypes have better control of CO_2_ diffusion mechanisms than those that are less tolerant to this stress. Therefore, the aim of this study was to evaluate the differences in the photosynthetic processes between two contrasting quinoa ecotypes, as well as to analyze the relative importance of non-diffusional factors in the two ecotypes when subjected to salinity conditions.

## 2. Materials and Methods 

### 2.1. Plant Material

The two quinoa ecotypes used in this study, Amarilla and Hueque, have been previously characterized as tolerant and sensitive to salinity, respectively [30]. The Amarilla ecotype comes from the salt flat area in the northern highlands, and Hueque originates from the wet area at sea level in southern Chile.

Disinfected seeds (2% sodium hypochlorite for 7 min) were germinated in 330 mL pots using perlite as the substrate. Pots containing 4 plants each were arranged over a tray containing water or solution nutritive and connected to it by means of a cotton wick to ensure water supply by capillarity. The plants were watered with sodium-free nutrient solution or 0 M (0.25 dS m^−1^) until the third pair of leaves developed (45 days after sowing (DAS)). At this stage, half of the pots of each ecotype were transferred to a modified Hoagland 2 solution containing 0.4 M NaCl (38.1 dS m^−1^). This concentration corresponds to the LD_50max_ for quinoa, as determined previously by Delatorre-Herrera and Pinto [30]. To avoid a saline shock, 0.4 M NaCl was applied in increments of 0.1 M per day. Control plants were irrigated with solution nutritive in a semi-hydroponic system (Schlick and Bubenheim [35], modified by Delatorre and Pinto [30]); for this purpose, filtered water with an electrical conductivity of 0.25 dS m^−1^ with a pH of 6 was used. When the plants developed their fourth or fifth pair of true leaves (70–75 DAS), they were placed in a shaded field, where the average temperature of the day was 20 ± 5 °C, and the maximum light intensity was 1500 µmol m^−2^s^−1^ of photosynthetically **a**ctive **r**adiation (PAR).

### 2.2. Na^+^ and K^+^

Sodium (Na^+^) and potassium (K^+^) contents were determined in samples of root, stems, and leaves taken from the bottom, middle, and top parts of the stem. Once collected, the material was dried at 70 °C for 48 h and then finely ground. Na^+^ and K^+^ were extracted by digesting 0.1 g of each sample in 15 mL of 0.5 M HCl for 2 days. The concentrations of Na^+^ and K^+^ were determined according to the procedure described by Hunt [36] using a flame photometer (Jenway Model PFP 7, Cole Parmer, Vernon Hills, IL, USA).

### 2.3. Proline and Glycine Betaine

Proline (Pro) content was determined spectrophotometrically (Spectrophotometer, Genesys, Thermo Scientific, Waltham, MA, USA) in tissue with a dry weight of 100 mg using the method described by Bates et al. [37]. A standard curve was constructed with proline (M.W. 115.13 g mol^−1^, Sigma-Aldrich, Santiago, Chile). 

The content of Glycine Betaine (GB) was determined according to Grieve and Grattan [38] but modified for quinoa. Briefly, tissue with a dry weight of 100 mg was stirred in 4 mL of water for 24 h at 25 °C, after which it was filtered and stored at 4 °C until analysis. For the determination of quaternary compounds, 50 µL samples were thawed, diluted in 50 µL of 2 N sulfuric acid, and cooled to 0 °C for 1 h. Then, 40 µL of KI-I_2_ reagent (15.7 g iodine and 20 g KI in 100 mL of water) was added and gently stirred in a vortex. This solution was stored at −4 °C for 16 h, after which it was centrifuged at 10,000 rpm for 15 min. The supernatant was carefully removed, and the precipitate was dissolved in 1.6 mL of 1,2-dichloroethane, shaken vigorously by vortexing, and left to stand for 2.5 h at room temperature. GB content was detected at 365 nm. The standard curve was constructed with betaine (M.W. 117.15 g mol^−1^, Sigma-Aldrich, Santiago, Chile).

### 2.4. Determination of CO_2_ Assimilation 

#### Gas Exchange

Gas exchange measurements were performed between 09:00 and 15:00 using a gas exchange chamber connected to a portable infrared analyzer (Licor 6200, LI-COR, Lincoln, NE, USA). The relative humidity of the chamber ranged from 40% to 50%, and the leaf temperature was 25 ± 2 °C. Measurements were made on fully expanded mature leaves (middle part of the stem). Each measurement was repeated three times on the same leaf (one leaf per plant and 6 plants per treatment). These measurements were taken when 50% of the plants had grown the fourth or fifth pair of true leaves (70–75 DAS).

(i) A/PFD curves

The photon flows (PFDs) used were 0, 20, 90, 120, 150, 300, 500, 700, 1200, 1500, and 2500 µmol photons m^−2^s^−1^. The light source was a halogen lamp, and the different PFDs were obtained by placing neutral filters between the lamp and the photosynthetic chamber.

The temperature in the photosynthetic chamber was 25 ± 2 °C. During measurements, the CO_2_ and the O_2_ concentrations in the chamber were maintained at 360 µL L^−1^ and 20%, respectively (CO_2_ and O_2_, certified gas, INDURA S.A, Alto Hospicio, Chile).

The gross photosynthesis rate (A), the apparent quantum efficiency (Φ), photosynthetically active radiation (PAR), the rate of assimilation at saturating light intensity (A_max_), and mitochondrial respiration in darkness (Rd) were obtained from the A/PFD curves adjusted to a non-rectangular hyperbole-type equation, according to the procedure described by Lambers et al. [39]. The straight section of the curve was adjusted to a polynomial equation whose intersection on the *x*-axis corresponds to the light compensation point, and the slope is the apparent quantum yield.

(ii) A/C_i_ Curves

Measurements were made at light saturation (1500 µmol photons m^−2^s^−1^). The relative humidity of the chamber ranged from 40% to 50%, and the leaf temperature was 25 ± 2 °C. The CO_2_ concentrations used were 50, 350, 500, 700, 900, 1200, and 1500 µL L^−1^, and the assimilation response to these intensities was adjusted to the Farquhar model [40,41] with PHOTOSYN software (version 1.1.2, Dundee Scientific Ltd., Dundee, UK). Parameters such as the maximum rate of RubisCO activity (V_c,max_), the transport of electrons at light saturation (J_max_), and the triose phosphate transport rate (TPU) were obtained from this model, which expresses the ratio of the assimilation rate (A) to the internal concentration of CO_2_ (C*_i_*) for each of the three factors affecting assimilation.

When carboxylation is limited only by the activity of RubisCO, then W_c_ can be described by the model developed by Farquhar et al. [40], which is based on gaseous exchange measurements.

When the transport of electrons limits photosynthesis due to the effect of RuBP regeneration, W_j_ can be expressed according to Farquhar and Von Caemmerer [41]. The potential rate of electron transport (*J_p_*) was calculated using the expression developed by Harley et al. [42].

(iii) Determination of the linear transport rate of electrons

To measure photosystem II (PSII) efficiency, the IRGA 6200 chamber was adapted by introducing a Hansantech PEA modulated pulse fluorometer sensor at one end. The parameters were calculated according to the methodology described by Maxwell and Johnson [43].

Quantum yield (ΦPSII) can be used to calculate the linear transport rate of electrons (*J*), which corresponds to photosynthetic capacity in vivo, according Genty et al. [44]. A factor of 0.84 is assumed for leaf absorbance in C3 plants [45], although this absorbance may change slightly with temperature (this experiment was conducted at foliar temperatures of 25 ± 3 °C).

The photochemical energy used for photosynthesis or photochemical quenching (qP) and non-photochemical quenching (NPQ) were calculated using equations described by Genty et al. [44].

### 2.5. Removal of Stomatal Effects

To remove the stomatal effect on CO_2_ assimilation (A), the methodology described by Centritto et al. [15] was used. For this purpose, a set of A/C_i_ curves were generated for both ecotypes. The procedure employed can be divided into four phases: In phase (I), stomatal restriction (R) was induced by lowering the concentration of CO_2_ from 350 to 50 µL L^−1^ and allowing g_s_ to decrease to values close to 15 mmol m^−2^s^−1^. Subsequently, in phase (II), the CO_2_ concentration was raised to 1500 µL L^−1^, and measurements of the A/Ci curve were made until 50 µL L^−1^. In phase (III), a CO_2_ concentration of 50 µL L^−1^ was maintained for a minimum of 40 min, which depended on the stomatal behavior of each ecotype and treatment; in this way, stomatal opening was activated. Phase (IV), the final phase, was initiated once stomatal conductance began to rise above 600 mmol m^−2^ s^−1^. This raised the CO_2_ concentration to 1500 µL L^−1^, and A/C_i_ was measured with no stomatal restrictions (nR). A value of 600 mmol m^−2^ s^−1^ was used based on the data of Jacobsen et al. [26], who reported a g_s_ of 600 mmol H_2_O m^−2^ s^−1^ in quinoa without water restrictions. The times used in each stage are detailed in Table 1.

### 2.6. Determination of Mesophyll Conductance (g_m_)

Mesophyll conductance (g_m_) was determined according to the procedure described by Harley et al. [42] using the following parameters: assimilation rate (A), light compensation point (Γ), electron transport rate (J), and dark respiration (Rd).

### 2.7. Rate Carboxylation and Electron Transport In Vivo

The RubisCO activity rate at RuBP saturation (V_c,max_), the maximum electron transport rate (J_max_), the maximum rate of assimilation at saturating PFD, and CO_2_ (A_max_) and mitochondrial respiration in light per unit of foliar area (R_d_) were obtained using the model described by Farquhar et al. [40]. From this model, A was determined using Equation (1):

A = vc − 0.5vo − Rd(1)

where vc and vo are the rates of carboxylation and oxygenation of RubisCO, respectively, and 0.5 is the stoichiometric relationship between the O_2_ absorbed by RuBP oxygenase and the photorespiratory evolution of CO_2_ [41]. Additionally, vc cannot be greater than the minimum rate of carboxylation of RubisCO (A_c_), and the rate of electron transport limits the regeneration of RuBP (A_j_).

### 2.8. Data Analysis

A 2 × 2 factorial design was used, arranged in completely randomized blocks (B_i_). The first factorial level was NaCl concentration (C_j_) (0 M and 0.4 M NaCl), and the second level was the ecotype (A_k_) (Amarilla and Hueque). Each treatment had 6 replications, and the experimental unit consisted of 2 pots, each with 4 plants (8 plants). Duncan’s multiple comparison one-way analysis of variance (ANOVA) was performed using Infostat V 2016 (Universidad de Cordoba, Cordoba, Argentina) *p* < 0.01 was considered significant.

## 3. Results

### 3.1. Stress Indicators

**Figure 1:**
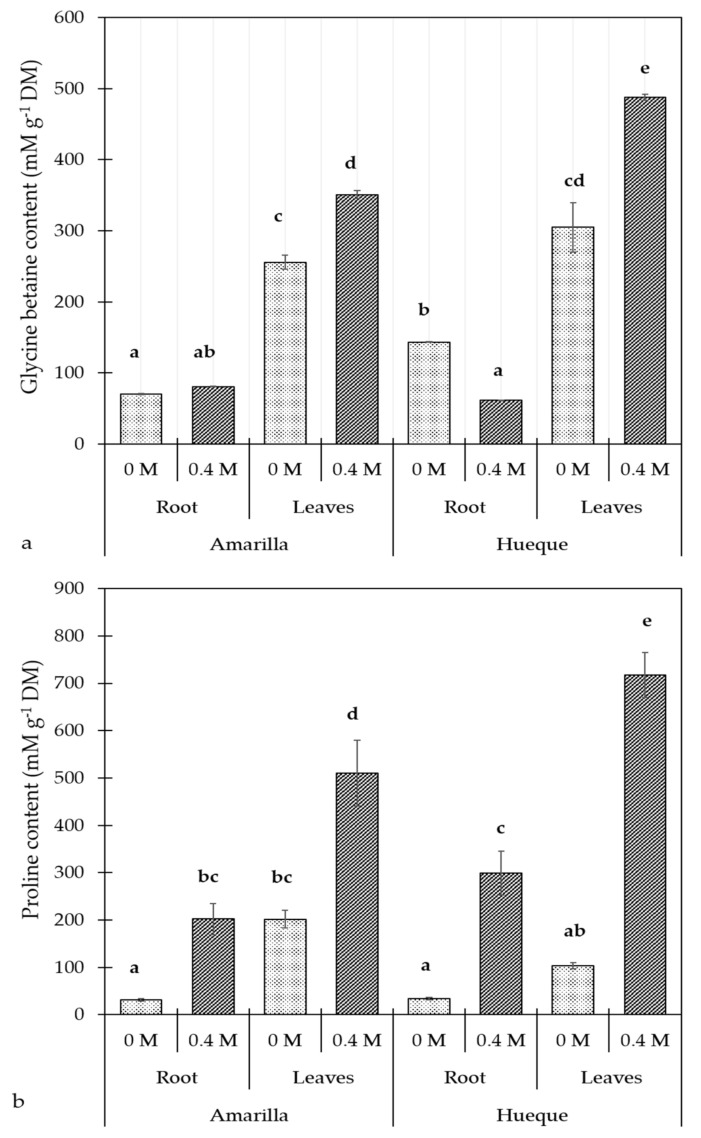
Effect of salinity on glycine betaine (**a**) and proline (**b**) content in the roots and leaves of quinoa plants. Different letters denote significant differences (*p* ≤ 0.05). Average values were calculated based on 4 samples per treatment (mean ± SE).

When comparing the control plants of the two ecotypes, Hueque had a higher concentration of GB in the roots. When exposed to 0.4 M NaCl, the GB content increased in both ecotypes, and it was higher in Hueque. Figure 1a shows that in the salt-tolerant ecotype (Amarilla), the GB content was higher in the leaves than in the roots. In the roots, salt stress had no effect on GB content, whereas in the leaves, GB content increased significantly. The leaf/root ratio of GB content in Amarilla control plants was 3.64, while in stressed plants, it rose slightly to 4.34, which could indicate that this ecotype is not severely stressed by high salinity. In the salt-sensitive ecotype (Hueque), the constitutive concentration of GB was also higher in the leaves than in the roots. In the roots, the salt stress caused a decrease in the GB content; in contrast, GB rose significantly in the leaves. The leaf/root ratio of GB was 2.12 in Hueque control plants and 7.95 in stressed plants, which means that the GB synthesis in leaves was more sensitive to salt signals coming from roots.

The Pro content in the control plants did not differ much between the two ecotypes. Exposure to 0.4 M NaCl increased the Pro content in all tissues tested in both ecotypes, and it was highest in Hueque. Figure 1b shows that in the tolerant ecotype, the content of Pro was significantly higher in the leaves than in the roots under both control and saline conditions. The leaf/root ratio of Pro in control plants was 6.53, and in salt-exposed plants, it fell to 2.53; this decrease was mainly due to the Pro increase in the roots. In Hueque, the content of Pro was greater in the leaves than in the roots. Applying salt did not cause changes in Pro levels in the roots, whereas in leaves, the Pro content rose significantly. The leaf/root ratio of Pro in control plants was 3.02, and when exposed to 0.4 M NaCl, it was 2.40.

### 3.2. Na^+^ and K^+^ Content

**Figure 2:**
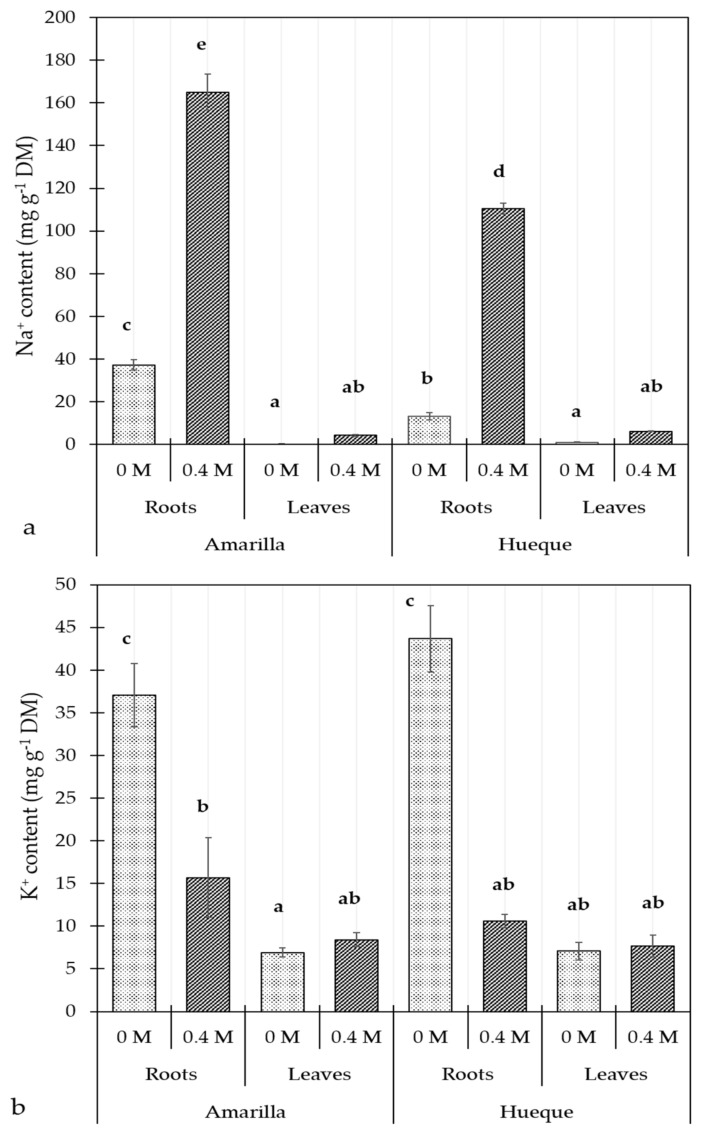
Effect of salinity on the content of Na^+^ (**a**) and K^+^ (**b**) in Amarilla and Hueque ecotypes of quinoa. Different letters denote significant differences (*p* ≤ 0.05). Average values were calculated based on 4 samples per treatment (mean ± SE).

The tolerant ecotype accumulated in root 64.6% more Na^+^ than the sensitive one in control conditions. Under salt stress, this difference decreased to 33% in both ecotypes because the sensitive ecotype accumulated proportionally more sodium than the tolerant one (7.4 versus 3.4 times, respectively) (Figure 2a). However, the plants showed no changes in the leaf Na^+^ level, regardless of salt exposure. The K^+^ content in roots was similar in the control plants of both tolerant and sensitive ecotypes and decreased significantly under salt stress. Under high concentrations of soil salt, the K^+^ content decreased in the roots and was maintained in the leaves in both ecotypes (Figure 2b).

### 3.3. Effect of Salinity on CO_2_ Assimilation under Different Light Intensities 

**Figure 3:**
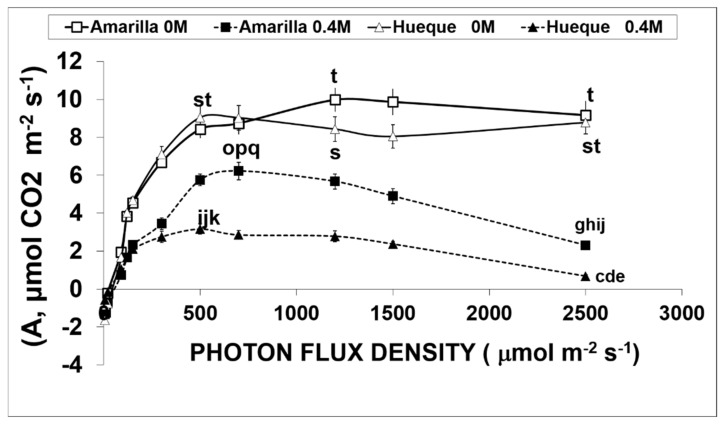
CO_2_ assimilation curves of two quinoa ecotypes subjected to salinity conditions. Empty symbols and continuous lines correspond to treatments without salt, while filled symbols with dotted lines correspond to 0.4 M NaCl treatments. Different letters denote significant differences (*p* ≤ 0.05). Average values were calculated based on 3 leaves taken from 6 plants per treatment (mean ± SE).

The A/PFD curves are shown in Figure 3. In the absence of salt stress, both ecotypes had a similar quantum yield performance and assimilation rate at light saturation. The A_max_ values for Amarilla and Hueque were 9.98 and 9.05 µmol CO_2_ m^−2^s^−1^, respectively (Table 2). However, the intensities at which light saturation reached were significantly different between the ecotypes: CO_2_ assimilation saturated at 957 PAR µm^−2^s^−1^ in Hueque and at 1262 µmol PAR m^−2^s^−1^ in Amarilla (Table 2).

Under saline conditions (0.4 M NaCl), both ecotypes presented a drastic decrease in CO_2_ assimilation rates with respect to control plants (Figure 3). For example, the decrease in A_max_ was 37.7% in the tolerant ecotype but 65.1% in the sensitive one (Table 3). On the other hand, at light intensities exceeding the corresponding light saturation point, saline conditions induced photoinhibition in both ecotypes. This was very drastic in the sensitive ecotype, in which assimilation was almost null at 2500 µmol photons m^−2^s^−1^ (Figure 3).

The analysis of the main photochemical parameters (Table 3) shows that the photochemical efficiency (ΦPSII) and rate of linear electron transport (J) were negatively affected by salt stress in both ecotypes. ΦPSII decreased by 18.1% for Amarilla and 29.2% for Hueque. J changed by 17.4% and 27.7% for Amarilla and Hueque, respectively.

### 3.4. Determination of Non-Restrictive (nR) and Restrictive (R) Stomatal Conductance

**Figure 4:**
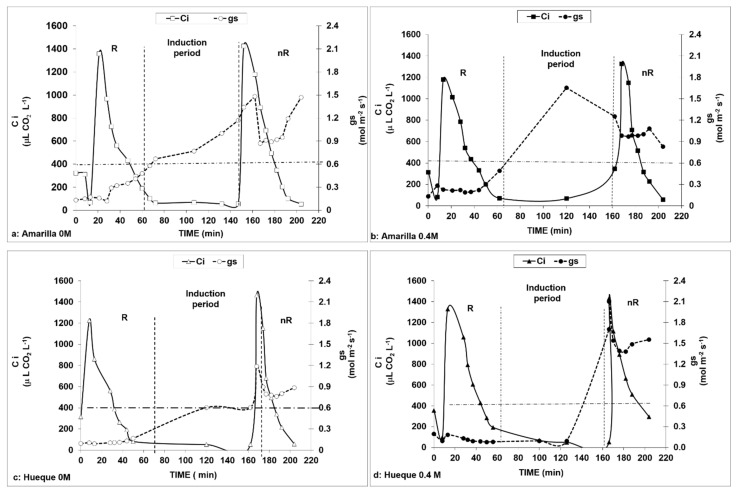
Curves of each ecotype used to determine the times necessary to induce stomatal opening. The continuous line represents the internal concentration of CO_2_ (Ci), and the dotted line is stomatal conductance. The line parallel to the *x*-axis represents the minimum limit required for open stomata. Lines parallel to the *y*-axis define the measurement periods. R—measurements with stomatal constraints; nR—measurements with no stomatal constraints. The induction period corresponds to the time interval required to induce stomata opening by applying a concentration of 50 μL CO_2_ L^−1^. Internal concentrations correspond to IRGA measurements with respect to the application of a specific environmental CO_2_ concentration, applied according to the time sequences described in Table 1.

Figure 4 shows the evolution of Ci and g_s_ at different timepoints, CO_2_ concentrations, and NaCl treatments for both ecotypes according to the procedure proposed by Centritto et al. [21]. This enabled us to determine the times and CO_2_ concentrations required to induce the rates of stomatal conductance, both restrictive (R) and non-restrictive (nR).

Once the CO_2_ concentration was lowered to 50 µL L^−1^ (phase III), the stomata opened after approximately 1.5 h (90 min). This allowed similar stomatal conductance behaviors to be maintained between non-stress and 0.4 M NaCl conditions in both ecotypes. The maintenance of Ci at values equal to or less than 50 µL L^−1^ caused a rapid response in the tolerant ecotype in control and saline conditions (Figure 4a,b). Thus, the method initially reduced the conductance to values close to 0.1 mol H_2_O m^−2^s^−1^, which then increased to 1.5 and 1.6 mol H_2_O m^−2^s^−1^ in the tolerant ecotype (at 0 and 0.4 M NaCl, respectively). In Hueque, the initial value was 0.09 mol H_2_O m^−2^s^−1^, which then increased to 1.2 mol H_2_O m^−2^s^−1^ at 0 M NaCl and 2.1 mol H_2_O m^−2^s^−1^ at 0.4 M NaCl. It was also observed that Hueque presented a higher g_s_ as well as a greater duration of stomatal opening in saline conditions (Figure 4c,d).

These data are consistent with the assimilation rates observed in plants with stomatal restriction (R) and without stomatal restriction (nR) (Table 4). In the salt-tolerant ecotype under control and nR condition, A_max_ increased from 13.8 to 21.43 µmol CO_2_ m ^−2^ s^−1^, which means that, in this case, the elimination of the stomatal restriction accounted for 55.7% of the assimilation rate. This effect was repeated in plants subjected to saline stress under the nR condition, but in this case, stomatal restriction accounted for only 25.4% of the increase in A_max_ (Table 4).

In the sensitive ecotype under non-salt conditions, the elimination of stomatal restriction did not induce a significant increase in A_max_ (6.7%), which suggests that this ecotype involves another resistance mechanism, such as mesophyll resistance. However, when salt-sensitive plants were treated with salt, elimination of stomatal restriction induced a 41.2% increase in A_max_. In both ecotypes, elimination of stomatal restriction induced a decrease in gross respiration under both control and salt stress conditions (Table 4).

The analysis of carboxylation parameters (Table 4) showed that the carboxylation efficiency in Amarilla control plants under non-restrictive stomatal conditions (nR) was not significantly altered (*p* > 0.05). However, in salt-stressed plants with nR, carboxylation efficiency was reduced by 44.1% compared to that observed in control plants, which may be associated with the effect of mesophyll conductance (g_m_). In the salt-sensitive ecotype, salt stress caused decreases in carboxylation efficiency of 76% and 48% for R and nR, respectively.

On the other hand, the unaltered maximum rates of RubisCO activity (Vcmax) and Triose Phosphate Transport (TPU) (Table 5) indicate that, in the tolerant ecotype, the reduction in CO_2_ assimilation caused by salt was not due to RubisCO activity or the supply of inorganic phosphate to chloroplasts. Differently, Vcmax, TPU in Hueque fell by 36.6% and 17.9% in R and nR conditions, respectively, showing that CO_2_ assimilation was affected by these factors (Table 5).

The quantum efficiency of PSII (ΦPSII) determined by the ratio of variable fluorescence (FV) and maximum fluorescence (Fm) (FV/FM) did not reveal an effect of salinity on the photochemical components of photosynthesis in either ecotype subjected to salt stress (Table 6). This means that photosystems I and II were not damaged. Therefore, it is unlikely that the decreases in the assimilation of CO_2_ and TPU are affected by the supply of ATP or NADPH.

## 4. Discussion

### 4.1. Effect of Salt on Stress Indicators

With respect to stress indicators, the present results confirm those of the previous studies in quinoa using the same or other ecotypes under similar salt conditions [16,30,46]. Pro accumulation has been reported in several southern Chilean quinoa accessions in response to 300 and 450 mM NaCl; genotypes considered tolerant to salt stress accumulated 3–5-fold more Pro than control plants, while the more sensitive ones exhibited moderate increases [19,46,47,48]. Several reports on glycophytes and halophytes have indicated that accumulation of GB depends on the genotype and salt stress intensity, and a positive correlation between GB levels and salt tolerance has been reported [49,50]. Our results concur with these previous studies, as increases in the GB and Pro contents were observed in the leaves of both ecotypes in response to salt stress; however, in this case, it was the sensitive ecotype that presented a greater accumulation of both osmolytes. While many studies have indicated a positive relationship between GB and/or Pro accumulation and plant stress tolerance, some have argued that increases in these compounds are a product of, non- adaptive response to, salt stress [16]. In drought-stressed corn, increased ABA content was followed by an elevation in betaine aldehyde dehydrogenase (BADH) activity, leading to GB accumulation [51]. Moreover, BADH and P5CS genes, related to GB and Pro biosynthesis, respectively, were upregulated in response to treatments with salt or chromium combined with salt in quinoa [50,52,53]. In addition, exogenous applications of GB and Pro in crop species such as rice and tomato help mitigate the effects of environmental stresses, including water and salt stress [47], meaning that they may have a role in adaptive responses to abiotic stresses. The salt-sensitive ecotype (Hueque) had a higher constitutive concentration of GB, and under salt stress conditions, the GB content in leaves was significantly enhanced and was higher than that in Amarilla. Under saline conditions, GB biosynthesis in most plant species occurs in the chloroplast [48]. Thus, GB production may be overstimulated in leaves, thereby increasing the shoot/root ratio of GB, as observed in Hueque. In this salt-sensitive ecotype, high foliar levels of GB may contribute to the maintenance of growth by acting as a nitrogen donor [54], contributing to osmotic homeostasis [55], and protecting the plant from oxidative damage, in combination with Pro [56]. In the sensitive ecotype (Hueque), the situation seems to be similar at the root level because, while GB decreased, Pro increased to levels that were 50% higher than those in the tolerant plant, which maintained its GB content under salt stress. 

Our results for Na^+^ and K^+^ contents agree with those reported by Adolf et al. [3] in two contrasting quinoa varieties, salt-tolerant and -sensitive, namely, Titicaca and Utusaya, respectively. They found that the Na^+^ content in the leaves increased drastically in plants treated with 0.4 M NaCl, and the increase was more pronounced in the salt-tolerant plant (Titicaca) compared to the more sensitive one (Utusaya). The significant increase found in leaf Na^+^ content in these two genotypes could be due to the duration of salt treatment (42 days of salt treatment vs. 15 days in our experimental design). 

The salt treatment also caused a significant increase in the K^+^ concentration in the xylem of both ecotypes, but there were no differences in K^+^ between treated and untreated plants. Our findings are similar in that there were no differences in leaf K^+^ content between control and salt-treated plants. Our findings are similar to those reported by Orsini et al. [16] in that there were no differences in leaf K^+^ content between control and up to 450 mM NaCl salt-treated plants of the coastal ecotype accession BO78. Potassium ions are essential for enzyme activity, protein synthesis, photosynthesis, osmoregulation, transport of phloem solutes, and the maintenance of the cation–anion balance in the cytosol and vacuoles. Recently, K^+^ was also proposed to be a secondary messenger [57]. Thus, the ability of plants to retain K^+^ under salt stress has emerged as an important trait for salt tolerance. By contrast, Na^+^ is toxic in glycophytic plants, but not in halophytes [58]. Sodium ions cause multifactorial responses, such as osmotic stress [59,60], the inhibition of vital enzymes [61,62], and competition with K^+^. Under salt stress, accumulated ions, such as Na^+^, Cl^–^, and K^+^, are used for osmotic adjustment in the aerial portions of halophytic plants [63], thus facilitating water uptake and transport and, presumably, lowering the metabolic cost required to produce large amounts of organic osmolytes, as previously described by Hariadi et al. for other quinoa genotypes [31].

At the root level, both tolerant and sensitive plants had similar responses in salinity conditions, decreasing the uptake of K^+^ and increasing the absorption of Na^+^; the latter was especially pronounced in the tolerant ecotype (Amarilla). In some cases, Na^+^ can replace K^+^, particularly in its osmotic functions in vacuoles under K^+^ starvation conditions. Halophytes require less K^+^ for growth than glycophytes [1,10], demonstrating a link between the ability to replace K^+^ with Na^+^ and salt tolerance [64], which is consistent with the results found in this study, in which the salt-tolerant ecotype had higher levels of Na^+^. In barley, a high concentration of Na^+^ allows plants to osmotically adapt to and maintain turgor under high salinity, which is a metabolically inexpensive mechanism for osmotic adaptation. However, this new Na^+^/K^+^ homeostasis could create a greater demand for organic solutes for osmotic adjustment, thereby compromising the energy balance of the plant [65]. In salt-tolerant barley cultivars [66], K^+^ was reported to be the main contributor to cytoplasmic osmolality, whereas in salt-sensitive genotypes, GB and Pro compensated for reduced cytosolic K^+^ levels.

### 4.2. CO_2_ Assimilation and Stomatal and Mesophyll Conductance

In both ecotypes, the decrease in CO_2_ assimilation due to salinity was accompanied by a decrease in stomatal conductance. However, this decrease in g_s_ was greater in Hueque, (57%, Table 2), than in Amarilla, where was only 17.4%. These values are consistent with those reported by Centritto et al. in olive [15] and by Killi and Haworth in quinoa [2]. These authors concluded that the reduction in stomatal conductance in these species is the principal factor in reducing CO_2_ diffusion, inducing a decrease in the internal CO_2_ gas pressure and thus reducing the rate of photosynthesis. In Hueque, the magnitude of the decrease in g_s_ (57.3%) due to salt treatment was proportional to the decrease in net CO_2_ assimilation (65.1%). However, in Amarilla, reduction in CO_2_ assimilation (37.7%) was not accompanied by a proportional reduction in g_s_ (17.4%). Thus, assuming a direct relationship between CO_2_ assimilation and g_s_, in the tolerant ecotype, only 46% of the reduction in CO_2_ assimilation caused by salt can be explained by the reduction in stomatal conductance. By contrast, in the case of Hueque, 90% of the reduction in CO_2_ assimilation can be associated to the g_s_ reduction. This finding suggests that in the tolerant ecotype the CO_2_ assimilation rate depends less on g_s_ than in the case of the sensitive one. This in turn suggests that, in this ecotype, another diffusional mechanism may be involved in controlling CO_2_ assimilation, such as mesophyll conductance (g_m_) for example. This possibility is also supported by Bongi and Loreto [19], who indicated that g_m_ was reduced under saline stress conditions in olive.

Despite the different behaviors of the ecotypes, g_s_ was low in both when treated with NaCl, suggesting that Na^+^ did not interfere with K^+^ stomatal signaling or guard cell osmoregulation [61], which is consistent with the exclusion of Na^+^ from salt-stressed quinoa [62]. This concurs with the data shown in Figure 2, which shows that the K^+^ content of leaves did not change under saline conditions.

Calculations of mesophyll conductance (Table 7) in control conditions indicate that at 350 µL CO_2_ L^−1^ and light saturation, g_m_ was 57 mmol H_2_O m^−2^s^−1^ in Amarilla, representing 18% of g_s_, but was 38 mmol H_2_O m^−2^s^−1^ (< 8% g_s_) in Hueque. This indicates that in favorable conditions, the relative importance of g_m_ in CO_2_ diffusion is greater in the tolerant ecotype than in the sensitive one. However, with the application of saline stress, the g_m_ values were significantly reduced in both ecotypes, falling by 49% in Amarilla (57 to 29 mmol m^−2^ s^−1^) and 47% (38 to 20 mmol m^−2^s^−1^) in Hueque (Table 7). Nevertheless, these g_m_ values only account for 11% and 10% of g_s_, respectively, which shows that under salt stress conditions, in both ecotypes, stomatal conductance exerts much greater control over CO_2_ assimilation, and g_m_ is of marginal importance.

Table 7 also shows g_m_ values obtained under conditions without stomatal restriction. In both ecotypes and conditions (R and nR), the increase in salinity (0.4 M NaCl) caused a reduction in g_m_, results similar to those found by Delfine et al. [23] in spinach (*Spinacia oleracea*). However, after eliminating stomatal constraints under non-salt conditions, g_m_ increased in both ecotypes, but the change was more significant in the tolerant plants (61% in Amarilla versus 21% in Hueque). This is consistent with Delfine et al. [23], who demonstrated that g_m_ was not irreversible in olive, as we show here for quinoa [2]. On the other hand, under saline conditions and with stomatal restrictions, both ecotypes had g_m_ values that were similar to those obtained without stomatal restriction. This finding confirms that, in quinoa, g_m_ is not a relevant factor in determining the diffusion of CO_2_ in salt conditions.

### 4.3. Effect of Salinity on Non-Diffusional Parameters

**Figure 5:**
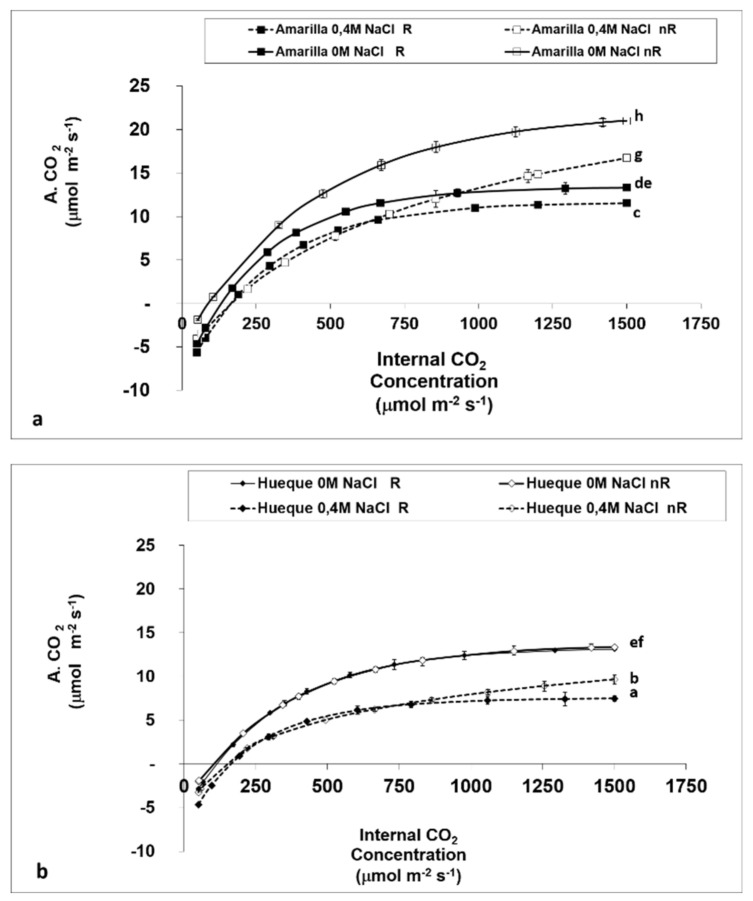
Evolution of CO_2_ assimilation based on internal CO_2_ concentration in the leaves of the quinoa ecotypes Amarilla (**a**) and Hueque (**b**) under limiting and non-limiting stomatal conditions. Different letters denote significant differences (*p* ≤ 0.01). Average values were calculated based on 3 leaves taken from 6 plants per treatment (mean ± SE).

The effect of salinity on CO_2_ assimilation, measured by the internal CO_2_ concentration and the effect of diffusion factors, is shown in Figure 5a,b. In both ecotypes stomatal limitations (R) strongly affected assimilation rates, especially upon the addition of 0.4 M NaCl. However, the ecotypes had different responses: while A increased in Amarilla, with the removal of stomatal constraints (nR), Hueque did not show the same response. This is similar to the observations reported in Table 2, which shows that Hueque has little control over stomatal opening mechanisms, which is apparently an important factor in decreasing the CO_2_ assimilation rate. However, above 900 µmol CO_2_, Amarilla had a CO_2_ assimilation rate in saline conditions similar to that of control plants in unrestricted conditions. Thus, the better response of Amarilla may be due to greater photochemical efficiency and greater RubisCO activity, while in Hueque, difficulties arise as a result of diffusion and lower energy efficiency (Table 5 and Table 6).

The results for CO_2_ assimilation in both ecotypes under restrictive (R) and non-restrictive (nR) conditions (Figure 4) indicate that, under restrictive conditions, there is little variation between salt-free and saline treatments in the tolerant ecotype (with conductance less than 300 mmol H_2_O m^−2^s^−1^). This result reaffirms earlier observations in this work: in the tolerant ecotype, the assimilation rate does not appear to be limited by stomatal conductance since A_max_ is only reduced by 7.2%. This is confirmed by the data of the A/Ci curve under non-restrictive conditions (Figure 3). The values show an increase of 55.7% in A_max_ without salt, whereas with salt, there is a smaller increase in the assimilation rate of 8.7% (Table 5). This result shows that the main limiting factor in the tolerant ecotype is non-diffusional. This finding, in addition to the resistance of the mesophyll, which contributes to the decrease in A, is consistent with the first conclusions of this study (Table 2).

Table 8 shows some photochemical parameters obtained from A/PFD curves: Quantum Requirement (QR), Light compensation points (LCP) and Rate of dark respiration (DR). The obtained values reveal that the quantum requirement rose with salinity, and the increase was greater in Hueque (168%) than in Amarilla (27%). This demonstrates that salt induced a decrease in the effectiveness of photosynthesis in the sensitive ecotype, which was caused by the increase in the QR and decrease in Jmax, Vcmax, and TPU. These results are similar to those found by Killi and Haworth [2], where salinity resulted in a lower Vcmax of RubisCO and a lower Jmax for the regeneration of RuBP.

Light compensation point (LCP) also differs between the two ecotypes. Hueque shows virtually no changes with the addition of salt, which together with decreased mitochondrial respiration, could reflect the absence of mechanisms to tolerate salinity. In contrast, the tolerant increased LCP and the mitochondrial respiration, which reflect that this ecotype increases its energy requirements to activate possible mechanisms that allow it to tolerate this environmental stress. The greater tendency towards photoinhibition and the lower LCP of Hueque, reveals an acclimatization to the lower light intensities typical from the southern part of Chile, compared to the high light intensities found in northern highlands where Amarilla is acclimated. To verify this assertion, several photochemical parameters were determined. The data show that control Amarilla and Hueque plants have statistically equal quantum efficiencies. While there is a tendency to decrease ΦPSII, salt application does not cause significant changes in quantum efficiency (FV/Fm) in either ecotype with respect to its respective control.

Table 5 shows the Jmax values found in Amarilla (120–152 µmol m^−2^s^−1^) are very similar to the average of 109 C_3_ species (134 µmol m^−2^s^−1^; [67]) but much higher than those found by Centritto et al. [15] in olive trees (79.5 µmol m^−2^s^−1^). Killi and Haworth [2] observed values from 72–152 µmol m^−2^s^−1^ in quinoa, and significant falls in saline conditions. In our study, the salt sensitive ecotype has the lower values (72–115 µmol m^−2^ s^−1^), demonstrating that in this ecotype exposure to saline stress causes reductions in Jmax, and that the decrease in A could also be associated with the transport of electrons. It is striking that comparing this parameter between 0 M and 0.4 M NaCl with and without stomatal restriction produces a significant reduction in Hueque, but not in Amarilla, indicating that the difficulties in the sensitive ecotype could be due to carboxylation, given by an effect on Jmax, which would indicate a problem on the supply of ATP and NADPH. According to Killi and Haworth [2], the reductions in Vcmax found in salt-stressed quinoa would be compatible with the altered carboxylation of RubisCO [68,69] and/or by the reduced content of RubisCO [70]. Salt stress also reduced the regenerative capacity of RuBP in quinoa indicative of reduced RuBP availability [69] and expression [71,72], particularly in salinity.

In the case of Hueque, the parameters evaluated highlight that in addition to the high incidence of g_s_ on the rate of CO_2_ assimilation, there is also an effect caused by RubisCO, which is seen by comparing carboxylation efficiencies and Vcmax, between salt and control growth conditions. Similarly, Jmax and the TPU also play a role.

The Vcmax values in Amarilla quinoa under control conditions found here were lower (29–33 µmol m^−2^s^−1^) than those reported by Wullschleger [67] (average values of 64 µmol m^−2^s^−1^ for 109 species) and Killi and Haworth [2] (values from 60 to 160 μmol m^−2^s^−1^ in quinoa). However, the decreases in Vcmax observed in plants sensitive to saline stress in our study, as well as the results of Killi and Haworth [2] in quinoa treated with salt, indicate that differences in reported values may be associated with the particular characteristics of the different ecotypes used. The tolerant plants had higher Vcmax values than sensitive ecotypes. In this regard, Manter and Kerrigan [73] indicated that, in woody species, the values ranged between 31.2 and 42.2 µmol m^−2^s^−1^ and were associated with low mesophilic conductance, similar to the two Chilean quinoa ecotypes used in our study. In addition, salinity also induced reductions in Vcmax and Jmax, corroborating the loss of photosynthetic ability. The reductions in Vcmax found in quinoa with salt stress are compatible with poor carboxylation by RubisCO [2,69,74].

The ability to maintain intact membranes and photosystems may enable Amarilla to maintain photosynthesis even in saline conditions. In the cells of photosynthetic organisms, salt stress leads to a decrease in cell volume, induces osmotic stress, and inhibits the photosynthetic electron transfer process [75,76,77], and in *Synechococcus* cells, PSII and PSI are both inactivated due to changes in the K^+^/Na^+^ ratio [76]. In our experiments, salt treatment led to a 10.5-fold reduction in the level of K^+^ in the roots of the salt-tolerant ecotype (Amarilla) and a 34.6-fold reduction in the more sensitive ecotype (Hueque). The lower supply of this element could mean that PSII is more inactivated in Hueque than in Amarilla, as shown by the CO_2_ assimilation rates (Table 2), resulting in high Na^+^ and Cl^−^ fluxes into cells, disrupting ion homeostasis, and leading to the accumulation of reactive oxygen species (ROS) [76,78,79], which is associated with membrane lipid peroxidation [80,81] and can adversely affect photosynthesis [81].

Control of oxidation is achieved through the synthesis of antioxidants such as polyphenols, which are divided into several subgroups, among which are the flavonoids (including flavonol glycosides and isoflavones) [82]. Tocopherol and carotenoids are known to be very important for the scavenging of lipid peroxides in *Synechocystis* 6803 [83]. Flavonol glycosides constitute the most abundant phenolics in quinoa seeds and leaves [84]. Several phenolic acids, including hydroxycinnamic acid and hydrobenzoic acid derivatives, have been identified in quinoa seeds and leaves [85]. Furthermore, the highest activity was observed in red-violet quinoa varieties containing both betacyanins and betaxanthins, with significant capacity/activity also exhibited by the yellow ecotype. These varieties or ecotypes are characterized by a high dopaxanthin content, whose dihydroxylated substructure is a powerful antioxidant [83]. Our results show that both ecotypes significantly increase the GB content as a mechanism to protect photosynthetic activity. Some plant varieties are able of biosynthesize GB, exhibiting a greater tolerance to abiotic stress, and often have enhanced growth and yield relative to varieties that do not accumulate GB [86]. The increased GB accumulation mainly occurs in the chloroplast and is responsible for initiating a network of interactions between the plant’s photosynthetic apparatus, its “stress” and “growth” hormones, and reactive oxygen species. The increased abiotic stress tolerance of plants able to accumulate GB appears in large part to be due to the ability of chloroplast-produced GB to protect the photosynthetic apparatus [86]. In particular, the accumulation of GB in the chloroplast in response to a stress signal protects enzymes and lipids that are required to maintain both the flow of electrons through thylakoid membranes and the continued assimilation of CO_2_ [87,88].

It is probable that the Amarilla ecotype also has other mechanisms for membrane protection, such as the presence of trehalose [89]. Trehalose can act as a structural component when incorporated into glycolipids, thereby stabilizing membranes [50,89,90].

## 5. Conclusions

The Calvin cycle requires energy inputs that come from the photochemical phase, and the data show that electron transport is not strongly affected in the tolerant ecotype, which had higher J and Jmax values than those found in the sensitive ecotype. This allows NADPH and ATP to maintain their contributions to the production of triose phosphates (TPU), which, according to our results, do not differ from the treatment without salt. This suggests that the Amarilla ecotype maintains its rate of RuBP under salinity conditions. On the other hand, the Quantum Requirement (QR), light compensation points, and the dark respiration rate are increased, which may be the result of an adaptation of the photochemical apparatus through membrane protection, as seen by the increase in GB.

In the sensitive ecotype (Hueque), the CO_2_ assimilation rate was affected in both the biochemical and photochemical components; in this respect, Vcmax, TPU, Jmax, J, ΦPSII, and QR dramatically decreased. Light compensation points and the dark respiration rate were not affected.

Another mechanism that was activated in the short term in response to salt treatment in both ecotypes was the exclusion of Na^+^ towards the leaves and growth centers. Both ecotypes retained Na^+^ in the roots and restricted its entry to the leaves. This mechanism must be associated with the compartmentalization and blocking of excess Na^+^ not only in the vacuoles of quinoa leaves (tissue tolerance) but also in those of root cells (ion exclusion), mechanisms that are important for protection in response to ionic toxicity induced by salt at the cellular level.

## Figures and Tables

**Table 1:** Times of application of different concentrations of environmental CO_2_ to induce the closure and opening of stomata.

Stage I		Stage II		Stage III		Stage IV	
Initial Period Closed Stomata		With Stomatal Restriction (R)		Induction Period Open Stomata		No Stomatal Restriction (nR)	
Time min	[CO_2_]	Time min	[CO_2_] µL L^−1^	Time min	[CO_2_] µL L^−1^	Time min	[CO_2_] µL L^−1^
0	350	21	1500	107	50	152	1500
8	350	28	1200	132	50	162	1200
13	50	32	900	147	50	167	900
		37	700			172	700
		47	500			177	500
		54	350			182	350
		60	200			187	200
		67	100			192	100
		72	50			204	50

**Table 2:** The effect of salinity on different photosynthetic values of Amax and g_s_ were obtained from curves of Figure 3 determined at the light saturation point and 350 µL CO_2_ L^−1^.

Ecotype	NaCl (M)	Saturation Intensity (µmoles PAR m^−2^ s^−1^)	Amax µmol CO_2_ m^−2^ s^−1^	% Fall	g_s_ mmol H_2_O m^−2^ s^−1^	% Fall
Amarilla	0	1262 c	9.98		310	
Amarilla	0.4	685 ab	6.22	37.7	256	17.4
Hueque	0	957 b	9.05		471	
Hueque	0.4	420 a	3.16	65.1	201	57.3

* Different letters indicate significant differences (*p* ≤ 0.01).

**Table 3:** Harvested energy efficiency (ΦPSII) and rate of linear electron transport (J).

Ecotype	NaCl	ΦPSII	Significance	J	Significance
Amarilla	0 M	0.27	c	167.6	c
Amarilla	0.4 M	0.22	b	138.4	b
Hueque	0 M	0.24	bc	147.9	bc
Hueque	0.4 M	0.17	a	106.9	a

* Different letters indicate significant differences (*p* < 0.01).

**Table 4:** The effect of salinity Amax, Gross Respiration and Carboxylation efficiency parameters of tolerant and sensitive ecotypes of quinoa, obtained from A/Ci curves (Figure 5).

Ecotype	NaCl	CONDITION	Amax		Sig.	Gross Resp.		Sig.	Carbox.		Sig.
	M		µmol CO_2_ m^−2^ s^−1^	± SD		µmol CO_2_ m^−2^s^−1^	± SD		Efic.	± SD	
Amarilla	0	R	13.8	1.03	cd	−4.09	1.39	ab	0.135	0.036	d
Amarilla	0.4	R	12.8	0.94	c	−4.8	1.15	a	0.117	0.034	d
Amarilla	0	nR	21.49	2.93	f	−2,9	1.12	cd	0.127	0.026	d
Amarilla	0.4	nR	16.05	2.46	e	−3.42	1.3	bc	0.071	0.033	b
Hueque	0	R	13.75	2.82	cd	−3.25	1.11	cd	0.096	0.026	c
Hueque	0.4	R	7.3	0.82	a	−3.2	0.31	cd	0.023	0.005	a
Hueque	0	nR	14.67	3.2	de	−1.95	0.95	e	0.07	0.027	b
Hueque	0.4	nR	10.31	1.62	b	−2.51	0.98	de	0.036	0.008	a

R: with stomatal restriction; nR no stomatal restriction; Amax: Maximum assimilation rate; Gross resp.: Gross respiration (dark and photorespiration); Carbox. Efic.: Carboxylation efficiency; Different letters indicate Significance level (*p* ≤ 0.01).

**Table 5:** The effect of salinity on the main photosynthetic parameters of tolerant and sensitive ecotypes of quinoa using the model of Farquhar et al. (1980).

Ecotype	NaCl	CONDITION	Jmax		Sig.	Vcmax		Sig.	TPU		Sig.
	M		μmol CO_2_ m^−2^ s^−1^	± SD	*p* ≤ 0.01	μmol CO_2_ m^−2^ s^−1^	± SD	*p* ≤ 0.01		± SD	*p* ≤ 0.01
Amarilla	0	R	152.66	62.62	d	32.76	6.41	c	10.56	2.77	d
Amarilla	0.4	R	120.53	20.61	c	29.75	4.57	bc	9.27	1.49	cd
Amarilla	0	nR	122.52	23.61	c	33.4	9.77	c	9.93	1.83	cd
Amarilla	0.4	nR	122.59	24.82	c	29.55	5.06	bc	9.69	1.57	cd
Hueque	0	R	115.15	24.66	c	27.68	4.94	b	8.96	1.83	bc
Hueque	0.4	R	72.29	13.55	a	18.86	2.72	a	5.68	1.26	a
Hueque	0	nR	94.66	21.29	b	25.67	6.55	b	7.88	2.07	b
Hueque	0.4	nR	75.67	17.51	a	19.26	5.62	a	6.47	1.74	a

R: with stomatal restriction; nR without stomatal restriction; Vc,max is: Maximum rate of RubisCO activity; Jmax is the transport of electrons at light saturation and TPU is triose phosphate transport rate. Different letters indicate Significance level (*p* ≤ 0.01).

**Table 6:** Quantum efficiency of PSII.

ECOTYPE	M NaCl	ΦPSII	Significance *
Amarilla	0	0.82	b
Amarilla	0.4	0.78	ab
Hueque	0	0.79	ab
Hueque	0.4	0.77	a

* Different letters indicate significant differences (*p* ≤ 0.01).

**Table 7:** The effect of salinity on mesophyll conductance (g_m_) determined at the light saturation point, with and without stomatal restriction.

Ecotype	NaCl (M)	With Stomatal Restriction (R)		No Stomatal Restriction (nR)	
		g_m_ mmol H_2_O m^−2^ s^−1^	% Fall	g_m_ mmol H_2_O m^−2^ s^−1^	% Fall
Amarilla	0	57		92	
Amarilla	0.4	29	49.0	32	65.2
Hueque	0	38		46	
Hueque	0.4	20	47.4	20	56.5

**Table 8:** Effect of salinity on the QR, LCP and DR of two quinoa ecotypes.

Parameters	Amarilla (Tolerant)				Hueque (Sensitive)			
	0 M	0.4 M	Difference	%	0 M	0.4 M	Difference	%
Quantum Requirement (QR) µmol photons/µmol CO_2_	35.2	44.6	9.43	27	28.7	76.9	48.27	168
Light compensation points(LCP) Photons	15.2	59.5	44.3	291	15.9	15.6	−0.30	−2
Rate of dark respiration (DR) µmol CO_2_	−0.432	−1.33	−0.898	208	−0.553	−0.202	0.351	−63

## Data Availability

The data presented in this study are available in this article.
